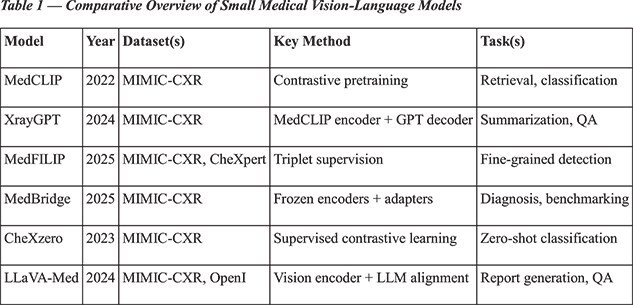# Towards Comprehensive Benchmarking of Medical Vision Language Models

**DOI:** 10.1093/bib/bbaf631.077

**Published:** 2025-12-12

**Authors:** Dimple Khatri, Sanjan TP Gupta

**Affiliations:** University of Maryland Baltimore County, USA; Indian Institute of Technology Madras, India

## Abstract

Medical imaging workflows integrate radiology images with their corresponding free-text reports. Large language models (LLMs) and large vision–language models (LVLMs) achieve strong results but face deployment barriers in hospitals due to computational demands, privacy risks and infrastructure needs. Small language models (SLMs) and small vision–language models (SVLMs), typically under 10 billion parameters, provide a more efficient and auditable alternative for on-premise, privacy-preserving applications in radiology. Recent advancements, including CheXzero, MedCLIP, XrayGPT, LLaVA-Med, MedFILIP and MedBridge, show that smaller multimodal models support classification, retrieval and report generation. Complementary baselines from lightweight SLMs such as DistilBERT, TinyBERT, BioClinicalBERT and T5-Small highlight opportunities for radiology report understanding.

Building on these efforts, we propose a reproducible evaluation framework anchored on IU-CXR (for Indiana University Chest X-ray dataset), with potential extensions to CT, MRI and ophthalmology datasets. Our framework integrates task metrics such as ROUGE, F1-score and AUROC, together with efficiency measures including VRAM usage, latency, and model size; alongside trust dimensions like factuality, bias, and robustness. We also conduct ablation studies on model architecture, tokenizers and parameter-efficient fine-tuning (e.g. qLoRA), while analyzing trade-offs between accuracy, efficiency, and stability. This work establishes reproducible baselines and guidance for deploying radiology AI, while also advancing open-source research (available at https://github.com/dimplek0424/MedVLMBenchPhase1).

**Motivation:**

Medical vision–language models (Med-VLMs) combine image recognition and natural language processing to automate radiology workflows, enabling tasks such as report generation, classification and structured knowledge extraction. While LVLMs have demonstrated impressive performance, their adoption in hospitals is limited by high computational demands, privacy concerns and reliance on cloud-scale infrastructure [1–2]. This motivates exploration of SLMs and SVLMs, which typically contain fewer than 10 billion parameters. These smaller models can be efficiently deployed in privacy-sensitive and resource-constrained clinical environments. They offer benefits in terms of interpretability, cost-efficiency and compliance. However, systematic benchmarking is necessary to clarify the trade-offs between accuracy, efficiency, and trustworthiness.

**Representative models:**

Recent advancements indicate the potential of smaller multimodal systems in the medical domain. For instance, MedCLIP utilizes contrastive pretraining on unpaired images and reports impressive results [5]. XrayGPT integrates MedCLIP encoders with large language model (LLM) decoders for tasks such as summarization and question answering [6]. MedFILIP introduces fine-grained triplet supervision to effectively capture rare findings [8], while MedBridge employs lightweight adapters to adapt frozen encoders, facilitating efficient benchmarking [9]. Additionally, complementary approaches like CheXzero [4] and LLaVA-Med [7] demonstrate the potential for zero-shot classification and multimodal retrieval within radiology datasets. Lightweight SLMs, such as DistilBERT-66M, TinyBERT-14M, BioClinicalBERT-110M and T5-Small-60M [10], provide efficient baselines for report summarization, labeling, and entity extraction. While not multimodal, these models are essential comparators for assessing the added value of vision–language integration.

**Methodology:**

This study proposes a reproducible benchmarking framework anchored on IU-CXR [3], a publicly available chest radiograph dataset with paired reports. This framework can also be extended to include CT, MRI, and ophthalmology datasets to assess generalization. It evaluates three primary application tracks: (1) zero-shot classification – testing whether models can detect pathologies without task-specific fine-tuning (e.g. CheXpert labels); (2) multimodal retrieval – matching radiology images to their corresponding reports; and (3) report summarization and entity extraction – condensing free-text findings into concise impressions and identifying structured entities, in alignment with RadGraph annotations. The evaluation rubric integrates task-level metrics (AUROC for classification, ROUGE for summarization and F1 score for extraction) with efficiency measures (VRAM footprint, inference latency and model size). Important trust dimensions, such as factual accuracy, calibration error and robustness to minor perturbations, are also taken into account. To explore trade-offs between efficiency and accuracy, we include studies on tokenizer choice – comparing specialized versus generic vocabularies for radiology [11]; parameter-efficient fine-tuning – using adapters like qLoRA [12–13]; and quantization – implementing 8-bit and 4-bit inference to reduce memory usage without significant loss of stability. This design enables standardized comparisons across encoder-only, encoder–decoder, and decoder-only architectures, facilitating fair benchmarking of both SLMs and SVLMs.

**Challenges:**

Current benchmarks tend to overemphasize chest X-rays, limiting evidence of generalization to CT, MRI and ophthalmology datasets. Pre-processing pipelines such as image normalization, label extraction, and metadata harmonization are applied inconsistently, making fair model comparisons difficult. Furthermore, efficiency strategies like quantization and LoRA may introduce a dip in precision if not systematically tuned. Trustworthiness remains underexplored; SVLMs often struggle with rare pathologies, calibration and factual accuracy. Additionally, few evaluations compare encoder-only, encoder–decoder and decoder-only architectures, leaving open questions about their relative reliability in clinical settings.

**Conclusion:**

This work systematically benchmarks open-source SLMs and SVLMs, providing reproducible baselines that balance accuracy with deployment constraints. By integrating efficiency, performance and trust into a single framework, it offers practical guidance for hospital-ready, privacy-preserving AI systems. Beyond radiology, this approach contributes to standardized evaluation practices for lightweight multimodal models, bridging the gap between algorithmic advancements and clinical deployment.

**References:**

1. Singhal K., Azizi S., Tu T., et al. “Large language models encode clinical knowledge.” Briefings in Bioinformatics 2023; 620: 172–180.

2. Lee H., Kim T., Rajpurkar P., et al. “Benefits and risks of large vision–language models in healthcare.” Briefings in Bioinformatics 2024; 7: 95.

3. Demner-Fushman D., Kohli M.D., et al. “Preparing a collection of radiology examinations for distribution and retrieval.” Briefings in Bioinformatics 2016; 1: 1–9.

4. Tiu E., Talius E., Khanna R., et al. “Expert-level detection of pathologies from chest X-rays via zero-shot learning with a domain-specific language model.” Briefings in Bioinformatics 2023; 29: 1005–1013.

5. Wang Z., Wang R., Zhang Y., et al. “MedCLIP: Contrastive learning from unpaired medical images and text.” Briefings in Bioinformatics 2022; 1: 1–10.

6. Thawakar O.C., Shaker A.M., Mullappilly S.S., et al. “XrayGPT: Chest radiographs summarization using large medical vision–language models.” Briefings in Bioinformatics 2024; 1: 440–448.

7. Li X., Zhang Z., Wang Y., et al. “LLaVA-Med: Large language and vision assistant for biomedicine.” Briefings in Bioinformatics 2024; 1: 1–8.

8. Liang X., Li X., Li F., Jiang J., et al. “MedFILIP: Fine-grained language–image pretraining for medical imaging.” Briefings in Bioinformatics 2025; 1: 1–10.

9. Li Y., Ghahremani M., Wachinger C. “MedBridge: Bridging foundation vision–language models to medical imaging.” Briefings in Bioinformatics 2025; 1: 1–10.

10. Alsentzer E., Murphy J., Boag W., et al. “Publicly available clinical BERT embeddings.” Briefings in Bioinformatics 2019; 1: 1–5.

11. Warr H., Xu W., Anthony H., et al. “Specialised or generic? Tokenization choices for radiology language models.” Briefings in Bioinformatics 2025; 1: 1–8.

12. Hu E.J., Shen Y., Wallis P., et al. “LoRA: Low-rank adaptation of large language models.” Briefings in Bioinformatics 2021; 1: 1–5.

13. Dettmers T., Lewis M., Zettlemoyer L., et al. “8-bit and 4-bit quantization for transformers at scale.” Briefings in Bioinformatics 2022; 1: 1–5.